# Patient safety from the perspective of quality management frameworks: a review

**DOI:** 10.1186/s13037-021-00286-6

**Published:** 2021-03-22

**Authors:** Amrita Shenoy

**Affiliations:** College of Public Affairs, School of Health and Human Services, Healthcare Administration Program, University of Baltimore, 1420 N. Charles Street, MD 21201 Baltimore, USA

**Keywords:** Quality management frameworks, IOM’s six aims for improvement, IHI’s Triple Aim, Patient safety, Patient centeredness, High‐quality clinical care

## Abstract

Patient safety is one of the overarching goals of patient care and quality management. Of the many quality management frameworks, Beauchamp and Childress’s four principles of biomedical ethics presents aspects of patient centeredness in clinical care. The Institute of Medicine’s six aims for improvement encapsulates elements of high-quality patient care. The Institute of Healthcare Improvement’s Triple Aim focuses on three aspects of care, cost, and health. Given the above frameworks, the present review was designed to emphasize the initiatives the system has taken to address various efforts of improving quality and patient safety. We, hereby, present a contemplative review of the concepts of informed consent, informed refusal, healthcare laws, policy programs, and regulations. The present review, furthermore, outlines measures and policies that management and administration implement and enforce, respectively, to ensure patient centered care. We, conclusively, explore prototype policies such as the Delivery System Reform Incentive Payment Program that imbues the elements of quality management frameworks, Hospital-Acquired Conditions Reduction Program that supports patient safety, and Hospital Readmissions Reduction Program that focuses on curbing readmissions.

## Background

The logistics of patient care and healthcare management revolve around many aspects of optimized high-quality care. The Joint Commission (TJC), Malcolm Baldrige National Quality Award (MBNQA), and The Magnet Recognition Program signify healthcare accreditation, performance excellence, and nursing excellence, respectively [1–3]. TJC is the recognized global leader of healthcare accreditation [4]. It is an independent not-for-profit organization that offers an unbiased assessment of quality achievement in patient care and safety [4]. MBNQA is the nation’s highest presidential honor for performance excellence [5]. The Magnet Recognition Program designates organizations worldwide where nursing leaders successfully align their nursing strategic goals to improve the organization’s patient outcomes [6]. In addition to the above healthcare recognition, the Institute of Medicine (IOM) categorizes aspects of care delivery with its six aims for improvement [7]. The Institute of Healthcare Improvement’s (IHI's) Triple Aim comprises of three aspects: improving the experience of care, improving the health of populations, and reducing per capita costs of healthcare.

We, hereby, present a synthesis of how the perspectives of biomedical ethics, six aims for improvement, and the Triple Aim converge into a focal point of preserving patient safety and promoting improvement in care delivery. The present review elaborates and explains the clinical and managerial roles inherent in the logistics of patient safety in emergencies and non-emergencies. The impetus here is to exemplify existing policies supporting patient centeredness while preserving the parameters that improve patient care, preserve quality, and promote patient safety.

As one of the cornerstones of high-quality healthcare, patient safety is intrinsic to all healthcare professionals. Clinicians are involved in direct patient care. However, does that imply that policymakers, leadership, and managers are separate and distinct components not involved in patient safety? The answer to the above question is not likely because these entities devise and enforce policies to preserve and augment patient safety in communities, institutions, and departments. At the macro-level, policymakers devise and recommend healthcare policies that at the micro-level, leadership, management, and clinicians enforce, adopt, and practice, respectively, at the point of patient care.

## Research questions and objectives

Past literature establishes quality management frameworks such as Beauchamp and Childress’s Principles of Biomedical Ethics, six aims for improvement and the Triple Aim. The above frameworks, broadly, capture the patient’s needs/preferences while aligning with improvement in care delivery. However, there are instances in which patients when presented in an unconscious or inebriated state cannot communicate their treatment preferences. Given the above case, the first research question is: what are some recourses that providers can choose to adopt as safe harbors while treating such patients? The second research question is: what are the practices that clinicians could potentially adhere when the patient consents or refuses to consent? As a close follow-up, the third research question is: what is the role of administration in implementing policies that fall outside the purview of already enforced laws? The objective of the present review is threefold. First, we aim to propose answers to the dos and don’ts that clinicians could potentially adopt in emergency and non-emergency cases, given the concepts of informed consent and informed refusal. Second, we attempt to explain how hospital leadership can best facilitate patient safety and manage risk while facilitating high-quality patient care. Finally, we explore prototype policies such as the Delivery System Reform Incentive Payment program, Quadruple Aim, Hospital-Acquired Conditions Reduction Program, and Hospital Readmissions Reduction Program which have been implemented more recently as systemic initiatives to preserve patient safety and promote measures in care delivery.

## Literature review

### Quality management frameworks preserving patient safety: an overview of three established frameworks

#### Beauchamp and Childress’s principles of biomedical ethics

Faculty in medicine and surgery have a substantial role in ethically creating a culture of safety via medical and surgical treatments for patients. In this context, four principles of biomedical ethics come into the picture. Those principles are autonomy, non-maleficence, beneficence, and justice [9]. The above four principles are the four pillars of medical ethics and form the basis of ethical practice in medicine and surgery. Some more aspects of biomedical ethics stemming from the above four principles are considered in ethical medical and surgical decision making [10]. A list of those additional aspects are as follows: [10].

- Truthfulness, Full Disclosure, and Confidentiality: On the one hand, truthfulness is not distorting facts while presenting information to the patient; full disclosure is accurately and completely informing the details of the patient’s medical condition. On the other hand, confidentiality is the principle of not revealing information about the patient’s medical condition to third parties [10].
- Autonomy and Freedom: Autonomy is the principle of providing the patient discretion, freedom, and independence to choose treatment preferences. This concept particularly comes into the spotlight in end-of-life hospice treatments and medical terminations of pregnancies [10].
- Beneficence is the principle of doing good and inflicting the least harm to the patient.

### The Institute of medicine’s six aims for improvement model

The Agency for Healthcare Research and Quality (AHRQ) Patient Safety Network expands upon the definition of prevention of harm as, “freedom from accidental or preventable injuries produced by medical care” [11]. Furthermore, the IOM introduced six aims for improvement in healthcare to meet the patient’s healthcare needs and preserve patient safety. Those six aims are as follows: [7].

- Safe: avoiding injuries to patients from the care that is intended to help them. Patient safety can be a system-wide approach when patients see measures adopted and practiced that create a safe environment [7].
- Efficient: avoiding waste including waste from equipment, supplies, ideas, and energy. Healthcare wastes are also in the form of defensive medicine, malpractice litigation, systemic complexities, and administrative fraud and abuse. Cost-effective care potentially supports efficiency in healthcare [7].
- Effective: providing services based on scientific knowledge to all those who could benefit. In this context, Evidence Based Medicine is incorporating scientific knowledge into treatment and procedure options [7].
- Patient-centered: providing care that is respectful of and responsive to the patient’s needs, preferences, and values. Delivery of care is considered to be patient-centered when the patient can choose certain aspects of care. This approach towards patient care prospectively ingrains elements of cooperation and collaboration [7].
- Timely: reducing waiting times and detrimental delays for both, recipients and providers of care. Waits and harmful delays potentially produce life threatening illnesses worsening quality outcomes throughout the continuum of a patient care [7].
- Equitable: providing care that is consistent and does not vary in quality based on personal aspects such as gender, ethnicity, geographic location, and socioeconomic status, etc. [7].

As per the IOM’s six aims for improvement, first, healthcare processes need to be safe which implies the provider makes an active attempt to ensure patient safety. Second, patient care prospectively needs to be aligned with recent developments to be potentially effective. Third, patient-centered care takes into consideration the patient’s culture, dietary and personal preferences incorporated into care delivery methods. The above concept plays an important role in end-of-life or hospice care provided to the elderly. Fourth, timeliness is providing and receiving care in a manner that reduces waiting times and delays. On the one hand, unforeseen wait periods may delay care and result in serious unintended harm to patients. On the other hand, the provision of timely care is essential to patient safety. Fifth, focusing on eliminating wastes and redundant processes could potentially help conserving resources and making care more affordable. Finally, providing equitable care is that which does not vary in terms of race, ethnicity, socioeconomic status, and income [7].

### The Institute of healthcare improvement’s triple aim model

The Institute of Healthcare Improvement’s (IHI’s) Triple Aim model synthesizes and incorporates aspects of care, cost, and health [8]. The IHI’s Triple Aim model involves the following three components: [8].

- Improving the experience of care: Implementing Hospital Consumer Assessment of Healthcare Providers and Systems (HCAHPS) and Consumer Assessment of Healthcare Providers and Systems (CAHPS) surveys are few of the many ways of recording patient experience of care [12, 13]. The National Practitioner Data Bank (NPDB), additionally, assists in promoting quality health care and deterring fraud and abuse within health care delivery systems [14].
- Reducing per capita costs of care: Cost of care could be reduced with the help of using generic drugs instead of brand name drugs for prescriptions, as an example [8].
- Improving the health of populations [8].

The IHI's Triple Aim is a framework that describes an approach with a threefold purpose. First, improving the experience of care regarding healthcare quality, second, decreasing per capita costs of care that aims at reducing wastes and variation in healthcare, and third, improving the health of populations. The IHI’s Triple Aim model has universal applications that cover medical treatment, surgical care, therefore, opening avenues to solve administrative complexities for preserving health and wellness in populations.

The first component of the Triple Aim, improving the experience of care applies to advances in medical technology making a positive impact in the patient experience of care [8]. The second component of the Triple Aim, reducing per capita costs of care, applies to implementing telemedicine and telehealth projects, as an example. Telemedicine brings to fruition, efficient and timely care when physicians may not be in the vicinity of the patient [8]. On the one hand, one of pros of telemedicine is the potential to enhance access to care. On the other hand, it introduces this concept to some practitioners and patients who have little to no experience with e-health. The third component of the Triple Aim, improving the overall health of the population applies to facilitating a combination of the above two aims. The IHI’s Triple Aim model, therefore, is a three-pointed framework in which the first two aims are intrinsic to the third aim, improving population health [8].

## Discussion

### The roles of clinical faculty and administration in patient safety: adoption and implementation of best practices in emergency and non-emergency cases

Emergency Medical Treatment and Active Labor Act (EMTALA) is a federal law that requires anyone coming to an emergency department to be stabilized and treated, regardless of their insurance status or ability to pay [15]. As per EMTALA, the patient has a right to be treated and clinicians are bound to provide treatment [15]. In this context, let us consider an example of an unconscious patient in the emergency department that does not culturally prefer receiving blood transfusions. In the above case, hypothetically, if the treating provider is not knowledgeable of the cultural preference of the unconscious patient and proceeds to revive the patient via a blood transfusion, then, was patient centered care provided? The answer likely lies in the provider’s assessment in the context of EMTALA. The assessment, first and foremost, relates to the binding duty of the clinician to provide care to every patient, especially in times of emergencies.

The dynamics of the above hypothetical scenario entirely changes in non-emergency situations in which patients can choose a provider to treat them; and reciprocally, even providers can choose whom to treat. The rationale behind this is the physician-patient relationship that specifies the terms and conditions of a physician-patient contract [16]. This legal relationship is based on contract principles because the physician agrees to provide treatment in return for payment in the presence of the contract [16]. The law usually imposes no duty on the physician to treat the patient in the absence of a physician-patient contract [16].

In the process of providing treatment, obtaining informed consent is the concept in which the clinician explains the proposed line of treatment, duration, benefits, risks of opting in as well as opting out of the treatment, alternatives to the proposed treatment with an opportunity to answer patient questions [17]. In 1914, an American judge Benjamin Cardozo composed the foundational principle of informed consent as, “Every human being of adult years and sound mind has a right to determine what shall be done with his own body; and a surgeon who performs an operation without his patient’s consent commits an assault for which he is liable in damages” [18]. An interesting aspect of treatment in non-emergency cases is when the patient does not agree to informed consent which brings forth the concept of “Informed Refusal” [19, 20]. A living will is an example of an informed refusal document in which the patient states his or her end of life preferences [21]. In the above case, the provider honors the patient’s end of life preferences and/or withholds treatment for the patient as specified in the living will.

The role of leadership is to enforce EMTALA and help clinicians' awareness of informed consent and informed refusal processes in organizations. Moreover, they ensure that providers implement the above policies regarding patient preferences. In medical cases that fall outside the purview of the already enforced laws, leadership can prospectively make rules but with caution that those rules are not against public policy.

### Macro-level healthcare programs focusing on patient safety: prototype policies

#### Delivery system reform incentive payment program: focusing on alignment with quality management frameworks

The Delivery System Reform Incentive Payment (DSRIP) program is one prototype policy that incorporates six aims for improvement and the Triple Aim model. DSRIP has multiple healthcare projects that improve health statuses incorporating numerous metrics and milestones in primary care, specialty care, chronic care, navigation and case management, disease prevention and wellness, and general categories [23, 24]. These projects are reimbursed by the State Department of Health in a systematic manner when adopted by healthcare institutions [22–26].

DSRIP’s framework involves four components: (1) Infrastructure Development, (2) Program Innovation and Redesign, (3) Quality Improvement, and (4) Improvement in Population Health in states where its projects are implemented [22–26]. In its third year of implementation, the Texas DSRIP program in the southeastern county region had about 172 projects in eight cohorts those being, primary care, emergency care, chronic care, navigation/case management, disease prevention and wellness, behavioral health/substance abuse prevention, and general.[22, 23, 25] Each cohort had a set number of projects that involve meeting patient care milestones and metrics, simultaneously incorporating IOM’s six patient care aims of medical care being safe, efficient, effective, patient centered, timely, and equitable [22–25].

DSRIP, with all its projects implemented in the adopted regions and counties has been measured to improve population health [25]. A metric of measuring improvement in population health within the DSRIP program was preventable hospitalization rate [24]. The decrease in preventable hospitalization rates may have been attributed to the inherent design and dynamics of the DSRIP policy [23, 24]. Those dynamics comprised of factors such as physician-administrator collaboration, mechanisms of incentive payments, types of measures for reporting outcomes in quality, and interplaying healthcare externalities [24]. In the adopted regions and counties, a statistically significant decrease in preventable hospitalization rates was observed when tested with an interrupted time series method [25].

There were two phases of the Texas DSRIP program, DSRIP 1.0 and 2.0. It was in DSRIP 2.0 that comprehensive Diabetes Care: eye exam metric improved by 16 % while Influenza immunization improved by 12 % in the latter [27]. Researchers Revere et al. have identified that in DSRIP 2.0, the metrics for Central Line Associated Bloodstream Infection (CLABSI) rates, Catheter Associated Urinary Tract Infections (CAUTI), and Surgical Site Infection (SSI) rates improved by 26 %, 10 %, and 9 %, respectively [27].

#### Quadruple aim framework: focusing on the evolution of the triple aim

The Triple Aim, formulated in 2008, drew focus on three aims which were based on care, cost, and health. Sikka and colleagues, in 2015, constructed a fourth aim, improving the experience of providing care. This was made to acknowledge the importance of physicians, nurses, and all employees in “finding joy and meaning in their work and in doing so improving the experience of providing care” [28]. At the core of the fourth aim is the experience of joy and meaning in providing care making it synonymous with acquiring accomplishment and meaning in their contributions. The Quadruple Aim has broad implications in theory and practice factoring inclusiveness in terms of all members in the healthcare workforce [28].

#### Hospital-Acquired conditions reduction program: focusing on patient safety

The Hospital-Acquired Conditions Reduction Program (HACRP) is a Medicare pay-for-performance program that supports the CMS’ long-standing effort to link Medicare payments to healthcare quality in the inpatient hospital setting [29]. HACRP focuses on specific conditions that the Centers for Disease Control and Prevention (CDC) National Healthcare Safety Network (NHSN) healthcare- associated infection (HAI) measures which are: [30] (1) Central Line Associated Blood Stream Infection (CLABSI), (2) Catheter Associated Urinary Tract Infection (CAUTI), (3) Surgical Site Infection (SSI) for colon and hysterectomy, (4) Methicillin-Resistant Staphylococcus Aureus (MRSA) bacteremia, (5) Clostridium Difficile Infection (CDI).

Additionally, eight Patient Safety Indicators (PSIs) included in the program comprise of: [31] (1) PSI 03 - Pressure Ulcer Rate, (2) PSI 06 - Iatrogenic Pneumothorax Rate (3) PSI 07 - Central Venous Catheter-Related Bloodstream Infection Rate, (4) PSI 08 - Postoperative Hip Fracture Rate, (5) PSI 12 - Perioperative Pulmonary Embolism or Deep Vein Thrombosis Rate, (6) PSI 13 - Postoperative Sepsis Rate, (7) PSI 14 - Postoperative Wound Dehiscence Rate, (8) PSI 15 - Accidental Puncture or Laceration Rate.

#### Hospital readmissions reduction program: focusing on patient safety

The Hospital Readmissions Reduction Program (HRRP) is a Medicare value-based purchasing program that reduces payments to hospitals with excess readmissions. The program supports the national goal of improving healthcare by linking payment to the quality of hospital care [32]. HRRP has a specific focus on the following conditions to reduce readmissions that in turn improve patient safety [32]. Those conditions are as follows: [32] (1) Acute Myocardial Infarction (AMI), (2) Chronic Obstructive Pulmonary Disease (COPD), (3) Heart Failure (HF), (4) Pneumonia (5) Coronary Artery Bypass Graft (CABG) surgery, and (6) Elective Primary Total Hip Arthroplasty and/or Total Knee Arthroplasty (THA/TKA) [32].

## Conclusion

The purpose of the present review was to analyze patient safety through the lens of the above quality management frameworks. We, specifically, illuminated policies and laws such as EMTALA, informed consent, informed refusal, and living will as examples. In emergency cases, the rules of EMTALA apply whereas in non-emergency cases, the same applies to obtaining informed consent from the patient. In the event the patient refuses treatment, documenting the informed refusal would be ideal. We underscored selective new prototype policies percolating from national policymaking to institutional levels with a focus on the initiatives the system has actively taken to preserve patient safety and promote improvement in care delivery.
